# Effects of Steaming on Chemical Composition of Different Varieties of Purple-Fleshed Sweetpotato

**DOI:** 10.3390/foods13193168

**Published:** 2024-10-05

**Authors:** Xia Jiang, Rong Zhang, Yanqiang Yao, Chaochen Tang, Bin Wang, Zhangying Wang

**Affiliations:** 1 Guangdong Province Key Laboratory of Crop Genetic Improvement, Crops Research Institute, Guangdong Academy of Agricultural Sciences, Guangzhou 510640, China; jiangxia19992024@126.com (X.J.); zr9362@126.com (R.Z.); yaoyanqiang2024@163.com (Y.Y.); tangchaochen1988@163.com (C.T.); 2 School of Food Science and Technology, Shihezi University, Shihezi 832000, China; binwang0228@shzu.edu.cn

**Keywords:** purple-fleshed sweetpotato, starch, anthocyanins, volatile organic compounds, correlation

## Abstract

Purple-fleshed sweetpotatoes (PFSPs) are rich in anthocyanins and are one of the health foods of interest. In this study, the effects of steaming on the anthocyanin, starch, soluble sugar, volatile organic compounds (VOCs) and pasting properties of nine PFSPs from China were investigated. The anthocyanin content of raw PFSP ranged from 9 to 185 mg/100 g. The total starch content decreased and soluble sugar content increased in all purple potatoes after steaming. Among the nine PFSPs varieties, Guangshu20 showed the greatest decrease in starch content (30.61%) and the greatest increase in soluble sugar content (31.12%). The pasting properties affected the taste of the PFSPs, with Shuangpihuang having the lowest peak viscosity (720.33 cP) and Guangzishu12 having the highest peak viscosity (2501.67 cP). Correlation studies showed that the anthocyanin content and pasting properties were negatively correlated with most of the sensory indicators, whereas the soluble sugar content of steamed PFSPs was significantly positively correlated with sweetness. A total of 54 VOCs were identified in this study, and aldehydes and terpenoids were the major VOCs in PFSPs. This study provides a theoretical basis for the processing of different PFSP varieties.

## 1. Introduction

Sweetpotato (*Ipomoea batata* [L.] Lam.) ranks fifth among the most important food crops in developing countries [1], with a production of 46.8 million tonnes in China, which ranks first in the world for sweetpotato production [2]. Sweetpotato is considered to be one of the healthiest foods in the world because of its low glycemic indicator and nutrients such as dietary fiber, minerals and vitamins [3,4]. Purple-fleshed sweetpotatoes (PFSPs) are popular because they are rich in anthocyanins and can provide health benefits such as anti-oxidation, anti-inflammatory, hypolipidemic and hypoglycemic activity [5,6]. The proportion of sweetpotato in table-stock sweetpotatoes also increases with the improvement of people’s awareness of healthy diet, especially in southern China, where the proportion of table-stock sweetpotato consumption was as high as about 60% [7].

PFSPs are usually eaten after cooking, and thermal processing will have a certain impact on the quality and aroma of PFSPs. Previously, we studied the metabolomic changes in Guangzishu 9 under different processing methods, and the results showed that the starch degradation of PFSPs under the steaming method was the highest, the production of soluble sugar was also the highest, and the content of volatile organic compounds was higher, which could maximize the retention of PFSPs’ non-volatile metabolites compared with other cooking methods [8]. In addition, there was some loss of anthocyanins in PFSPs after cooking, and steaming can better retain their anthocyanins [9,10]. Starch, soluble sugar and anthocyanins can affect the formation of PFSPs’ taste through texture or sweetness [11,12]. The sensory evaluation of different varieties of PFSPs may vary due to their different anthocyanin content, starch content, and amylose content. However, current studies have mainly been conducted on single PFSP varieties, and the relationship between quality and the sensory evaluation of PFSPs is not yet clear. Therefore, further studies are needed to investigate the changes in the chemical composition of multiple PFSP varieties after steaming and the differences in their sensory evaluations.

In this study, the effects of steaming on the anthocyanins, starch, soluble sugar and volatile organic compounds of different PFSP varieties were determined by traditional chemical methods combined with HS-SPME/GC-MS, and a correlation analysis was carried out, in combination with a pasting properties and sensory evaluation, to provide a theoretical basis for the future processing of PFSPs. This study provides a reference for screening excellent PFSP varieties and consumer selection.

## 2. Materials and Methods

### 2.1. Chemicals and Reagents

1-Heptanol (≥99.5%, GC), glucose and n-alkanes were procured from Sigma-Aldrich Co. (St Louis, MO, USA), while all solvents were of LC-MS grade. Anthrone, sulfuric acid, sodium hydroxide and potassium chloride were obtained from Guangzhou Chemical Reagent Factory. A Milli-Q Water Purification System (Millipore, Bedford, MA, USA) was utilized for the production of ultrapure water.

### 2.2. Plant Material

The varieties Guangshu 20 (G20), Jicheng4 (J4), Guangzishu 1 (GZ1), Guangzishu 2 (GZ2), Guangzishu 9 (GZ9), Guangzishu 10 (GZ10), Guangzishu 12 (GZ12) and Shuangpihuang (SPH) were developed by the Crops Research Institute of Guangdong Academy of Agricultural Sciences in Guangzhou, China. Zhanzishu 3 (ZZ3) was bred by Zhanjiang Academy of Agricultural Sciences in Zhanjiang, China. Nine PFSPs varieties were cultivated using standard agricultural practices in the experimental field of Zhanjiang Academy of Agricultural Sciences in Zhanjiang, China. The planting took place in July and harvesting occurred in December. Root samples were collected after a growth period of 150 days.

### 2.3. Cooking Methods and Sample Preparation

Six medium-sized PFSPs of the same variety were randomly divided into 2 groups. One group was used as the raw material sample, and the other group was steamed at 100 °C for 40 min. All samples were not peeled before the heat treatment. For the sample processing method please refer to Jiang et al. [8].

### 2.4. Determination of Color Characteristics of Purple-Fleshed Sweetpotato

The color characteristics were measured using a CM-700D spectrophotometer (Konica Minolta Sensing Americas, Tokyo, Japan), and each group of samples was measured three times [8].

### 2.5. Determination of Total Anthocyanin Content

The total anthocyanin content of the samples was determined using the pH difference method, following the protocols described by Jiang et al. [13], with some modifications. Briefly, 1.00 g of a freeze-dried powder sample was weighed and mixed with 10 mL of hydrochloric acid ethanol extract, which was then shaken for 10 s and subjected to ultrasonic oscillation for 10 min. Subsequently, an additional 5 mL of hydrochloric acid ethanol extract was added, followed by another round of shaking and ultrasonic oscillation. This process was repeated once more with another 5 mL of hydrochloric acid ethanol extract. The resulting mixture was centrifuged at 4000 rpm for 10 min at a temperature of 4 °C, and the supernatant was pipetted into a 25 mL volumetric flask and fixed in volume. Next, each solution (1 mL) was diluted to a final volume of 10 mL using pH-adjusted solutions: pH 1.0 hydrochloric acid–potassium chloride solution and pH 4.5 hydrochloric acid–sodium acetate solution, respectively. After thorough mixing, the solutions were refrigerated in darkness for equilibrium over a period of 30 min before measuring their absorbance values at wavelengths of 530 nm and 700 nm against distilled water as a control sample. The total anthocyanin content in each sample was expressed as milligram equivalents of Cyanidin-3-glu using the following formula:

(1) $$Anthocyanin\left(mg/100\;g\right)=\frac{A\times MW\times DF\times V\times 100}{\varepsilon \times L\times W}$$

A was the absorbance of the sample, A = (A530–A700) pH 1.0-(A530–A700) pH 4.5; MW was the molecular weight of Cyanidin-3-glu (449.2 g/mol). DF was the diluted multiple, DF = 10; V was the total volume of the extract (25 mL); ε was the molar extinction coefficient of Cyanidin-3-glu (29,600 L/cm.mol); L was the optical path of the colorimetric cup (1 cm); and W was the sample weight (1.0 g). All experiments were performed in triplicate.

### 2.6. Determination of Total Starch and Amylose

The total starch and amylose content was detected by the method described by Tang et al. [14] and Yao et al. [15]. The total starch and amylose of the samples were determined by Fourier Transform Near-Infrared (FT-NIR) spectroscopy (FT9700, PerkinElmer, Hopkinton, MA, USA) to establish a near-infrared (NIR) model of the samples. The NIR model was validated by determining the total starch and amylose content using the Total Starch Assay Kit (Megazyme, Wicklow, Ireland) and Amylose/Amylopecin Assay Kit (Megazyme, Ireland), respectively.

### 2.7. Determination of Soluble Sugar Content

The anthrone colorimetry method was adopted to determine the amount of total soluble sugar present x [8].

### 2.8. Determination of Pasting Properties

The pasting properties of freeze-dried powders of the samples were determined using a rapid viscosity analyzer (RVA) (Tech-master, PERTEN Newport Scientific, Stockholm, Sweden) and analyzed using TCW software [16]. The amount of sample used was 3.00 g and this was added to 25.0 mL of water. All samples were tested three times. The temperature changes in the tank during the stirring process were as follows: the sample was kept at 50 °C for 1 min and then raised to 95 °C (4 min) at a rate of 11.25 °C/min; it was maintained at 95 °C for 4.5 min; it was then cooled down from 95 °C to 50 °C (4 min) at a rate of 11.25 °C/min; finally, it was kept at 50 °C for an additional 3.5 min. The stirrer rotates initially at a speed of 960 r/min for the first 10 s and then remains constant at 160 r/min thereafter. The viscosity values are expressed in centipoise (cP).

### 2.9. Sensory Evaluation Analysis

The PFSPs were evaluated by 8 professional individuals following steaming and scored on a scale of 0–4 for firmness, aroma, sweetness, starchiness, viscosity, smoothness, coarse texture and fibrous texture. Additionally, an overall score ranging from 0 to 100 was comprehensively assigned. The sweetpotato evaluation standard was presented in Table S1. Prior to the study, written informed consent was obtained from all participants. All experiments were conducted in accordance with the regulations set forth by the Administration Committee of Guangdong Academy of Agricultural Sciences.

### 2.10. Determination of Volatile Organic Compounds (VOCs) by GC-MS

Volatile organic compounds were determined by GC-MS [8]. In brief, 1 g of a fresh sample was placed into a 20 mL headspace vial, and 2 mL of saturated NaCl solution was added. Then, 1 μL and 5 μL of 1-heptanol (0.137 μg/mL, CAS: 111-70-6) were added to raw and steamed samples as internal standards, respectively. The raw and steamed samples were incubated at 40 °C and 70 °C for 30 min, respectively. Then, manually sampled SPME scaffolds with DVB/CAR/PDMS fiber were extracted at the same temperature for 30 min. GC-MS analysis was performed on an Aglient 7890B gas chromatography system coupled with a 5977B mass spectrometer (Agilent Technologies, Santa Clara, CA, USA) and equipped with an HP-5 ms column (30 m, 250 μm, 0.25 μm) (Agilent Technologies, CA, USA). For specific procedures please refer to Jiang et al. [8].

### 2.11. Relative Odor Activity Value (rOAV) Analysis

The key aroma compounds of the PFSPs before and after steaming were determined by rOAV. When the rOAV ≥ 1, it can be identified as a key aroma compound, where rOAV = the relative concentration of volatile compounds/odor threshold in water [17].

### 2.12. Statistical Analysis and Visualization

The statistical software SPSS22.0 (SPSS-IBM, Chicago, IL, USA) was utilized for conducting a one-way analysis of variance (ANOVA) with a significance level set at 0.05. The visualization of the heatmap was performed using TBtools (Toolbox for Biologists; version 1.113, China), and correlation heat maps were analyzed using Origin 2022 (OriginLab Corporation, Northampton, MA, USA). An unsupervised principal component analysis (PCA) score plot was generated through the utilization of the Metware Cloud platform (https://cloud.metware.cn, URL (accessed on 7 July 2023)). All analyses were conducted in triplicate to ensure accuracy and all mapping data were standardized.

## 3. Results and Discussion

### 3.1. Phenotypic Investigation

**Figure 1:**
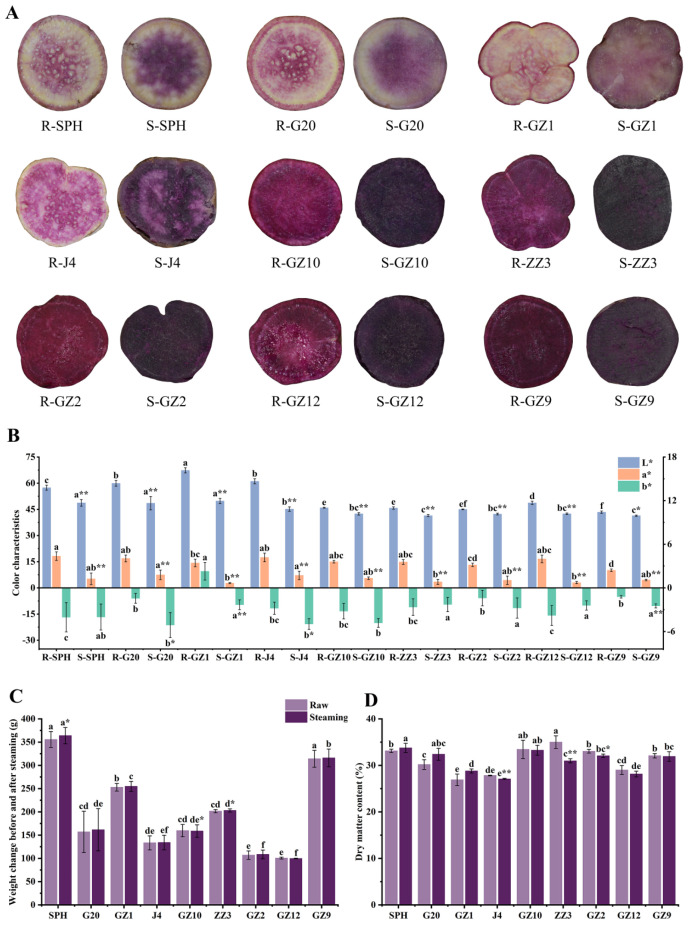
Phenotypic investigation of PFSPs before and after steaming was carried out. (**A**) Morphological observation of PFSPs before and after steaming. (**B**) The three color parameters (L, a*, b*) of PFSPs before and after steaming were detected by a portable colorimeter. (**C**) PFSPs’ weight change. (**D**) PFSPs’ dry matter content. Statistically significant differences based on *t*-tests. The different letters a, b, c, d, e and f indicate that there are significant differences in the results of different PFSPs. * indicates a significant difference between raw and steamed samples, ** indicates a highly significant difference between raw and steamed samples.

In order to gain a comprehensive understanding of the changes in PFSPs before and after steaming, we initially investigated their phenotypes (Figure 1A). The transverse section of their root tubers showed that SPH, G20, GZ1 and J4 had a mixed purple and white color (light PFSPs), while GZ10, ZZ3, GZ2, GZ12 and GZ9 were completely purple (deep PFSPs). The root color of the PFSPs changed after steaming (Figure 1B). The L* parameter of the light PFSPs is higher than that of the deep PFSPs, which may be due to the fact that light PFSPs contain another color, resulting in higher brightness. The parameters L* and a* of PFSPs were significantly reduced after steaming, indicating that steaming significantly reduced the brightness of PFSPs and the red color of PFSP tubers. The b* parameter was negative for all PFSPs except for GZ1, indicating that GZ1 was yellower than other PFSPs. The b* parameter of G20, GZ1, J4 and GZ9 decreased significantly after steaming, and the b* parameter of the remaining PFSPs did not change significantly. In general, there were significant differences in color between the different varieties and before and after steaming.

The weight of the PFSPs also changed after steaming (Figure 1C). The results showed that SPH’s weight significantly increased by 8.34 g after steaming, while the weight of the remaining PFSPs did not change much, indicating that the steamed PFSPs had better water retention [15]. In order to determine which factors caused the weight change, the dry matter content of the PFSPs was measured (Figure 1D). The highest dry matter content was found in ZZ3 (35.00%) of all the raw samples, while lower dry matter contents were found in GZ1, J4 and GZ12, which were all below 30%. The dry matter content of J4, ZZ3 and GZ2 decreased significantly after steaming, which may be due to the entry of water vapor into the PFSPs during steaming, and the remaining PFSPs did not show significant changes.

### 3.2. Total Anthocyanin Content

**Figure 2:**
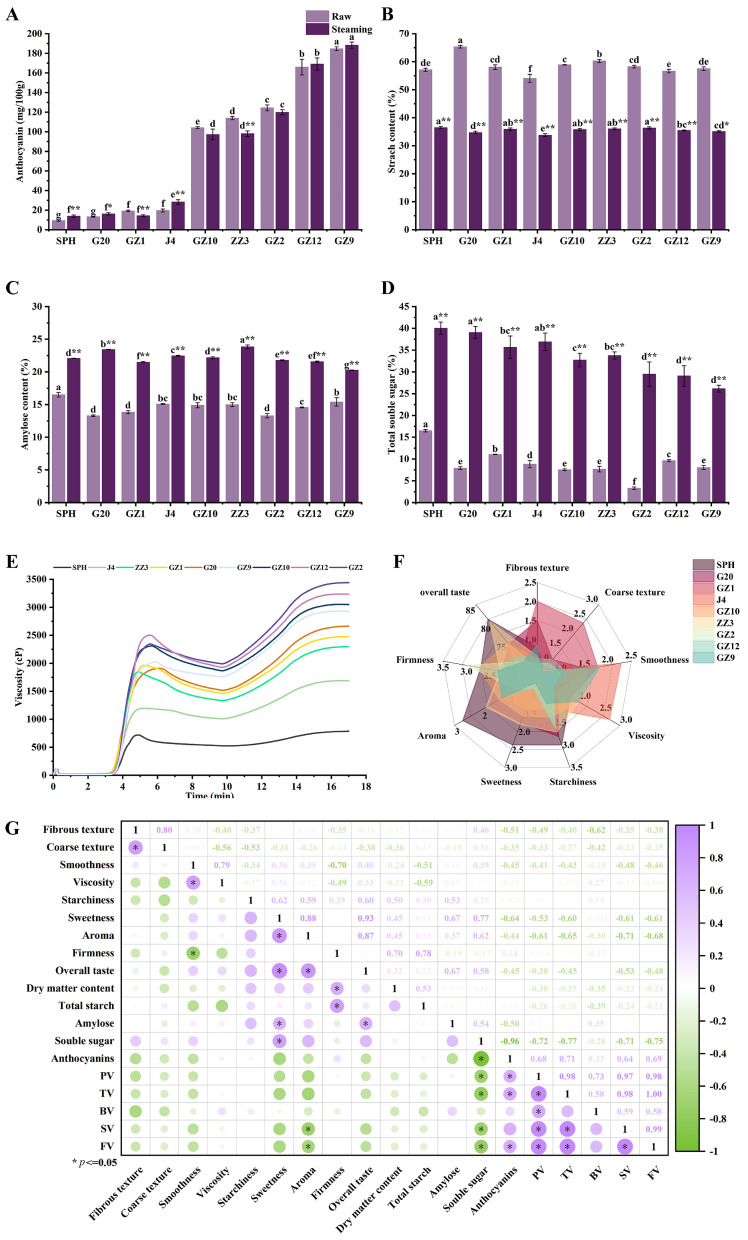
Investigation of basic physical and chemical indicators of PFSPs. (**A**) The total anthocyanin content of PFSPs before and after steaming. (**B**) The total starch content of PFSPs before and after steaming. (**C**) The amylose content of PFSPs before and after steaming. (**D**) The total soluble sugar content of PFSPs before and after steaming. (**E**) The pasting properties of different PFSPs. (**F**) The sensory evaluation of different PFSPs. (**G**) Correlation heat map of quality and sensory evaluation. Statistically significant differences based on *t*-tests. The different letters a, b, c, d, e, f and g indicate that there are significant differences in the results of different PFSPs. * indicates a significant difference between raw and steamed samples, ** indicates a highly significant difference between raw and steamed samples.

Anthocyanins are recognized as important bioactive substances in PFSPs, and their content and structure are influenced by the variety of sweetpotato, with the main anthocyanin monomers in PFSPs being anthocyanidins, peonidin and cyanidin [9,18]. The anthocyanin content affects the formation of their flesh color, and the anthocyanin content of light PFSPs is significantly lower than that of deep PFSPs (Figure 2A). In the raw samples, the anthocyanin content of deep PFSPs is about 5–11 times that of light PFSPs. The highest anthocyanin content was found in GZ9 (184.71 ± 1.93 mg/100 g) and the lowest was in SPH (9.37 ± 0.66 mg/100 g). After steaming, the anthocyanin content of GZ1 and ZZ3 decreased significantly, while that of SPH, G20 and J4 increased significantly, and the rest of the PFSPs’ changes were not significant. Most studies have shown that there is a loss of anthocyanin content after steaming, which is due to heat-induced enzyme inactivation and the disruption of cellular tissues, but some studies have shown an increase in anthocyanin content after steaming [9,19,20]. The increase in anthocyanins after cooking may be due to the increase in anthocyanin recovery after starch destruction and the inactivation of anthocyanins’ degrading enzyme by heating [9]. The trend in anthocyanin content after steaming was the same as that in the raw samples. A higher anthocyanin content, due to its bitterness, will affect the formation of sweetpotatoes’ taste [21], which is why most light PFSPs are less bitter than deep PFSPs.

### 3.3. Starch and Soluble Sugar Content

Starch and soluble sugar are important indicators in the sensory evaluation of sweetpotatoes which can affect the sweetness and aroma of sweetpotatoes. The total starch content of the raw samples ranged from 54.08% to 65.34%, with the highest being G20 and the lowest being J4. After cooking, the total starch content of all the PFSPs decreased by 20.30–30.61%, with the largest decrease in the total starch content of G20 (30.61%), followed by ZZ3 (24.25%), and the smallest decrease in J4 (20.30%). The reason for this is that the starch was gelatinized during steaming, and the starch was also degraded by amylase to produce soluble sugar [12]. The total starch content of light PFSPs was 33.78–36.46% and that of deep PFSPs was 35.09–36.34% after steaming. The amylose content of the PFSPs also changed significantly after steaming (Figure 2C). Among the raw samples, SPH had the highest amylose content of 16.50%, while G20 and GZ2 had the lowest, both at 13.30%. After steaming, the amylose content of all PFSPs increased significantly, among which G20’s amylose increased the most (10.13%), while GZ9’s increased by only 4.85%. The increase in amylose content may be due to the degradation of amylopectin’s branch structure during hot processing or the conversion of long-chain amylose to short-chain amylose [22]. 

The soluble sugar content of cooked sweetpotatoes is mainly positively correlated with sweetness. The soluble sugar content (dry weight) of all samples increased significantly after steaming (Figure 2D). Among the raw samples, SPH (16.49%) had the highest soluble sugar content, followed by GZ1 (11.07%), and the lowest was GZ2 (3.29%). After steaming, the soluble sugar content of light PFSPs was significantly higher than that of deep PFSPs. Among them, G20 showed the largest increase in soluble sugar content, and GZ12 and GZ9, which have a higher content of anthocyanin, showed the smallest increase, which was consistent with the results of previous studies and was mainly caused by the hydrolysis of starch under the heat treatment to produce large amounts of maltose [8,23].

### 3.4. Pasting Properties

The pasting properties of PFSPs are crucial to their sensory quality, especially texture properties [24,25], and their amylose content, starch granule size and so on affect their pasting properties [26]. The pasting properties of PFSPs are characterized by their RVA profile characteristics, that is, peak viscosity (PV), trough viscosity (TV), final viscosity (FV), breakdown viscosity (BV = PV − TV), setback viscosity (SV = FV − TV), paste temperature (PT) and peak time (the time required to reach the highest viscosity T). There were significant differences in the pasting properties of the nine PFSPs (Figure 2E, Table 1). Except for ZZ3, the PV (2024.00–2501.67 cP), TV (1758.67–1992.00 cP), FV (2929.67–3440.67 cP) and SV (1171.00–1448.66 cP) of the remaining deep PFSPs were significantly higher than the PV (720.33–1963.33 cP), TV (525.33–1518.00 cP), FV (786.33–2665.00 cP) and SV (261.00–1147.00 cP) of light PFSPs. The sensory quality of sweetpotato is a balance of individual pasting parameters and aroma. PV reflects the water-binding capacity of sweetpotato during starch pasting, which affects the viscosity of sweetpotato after cooking, and FV reflects the stability of the structure of the starch swelling granules. GZ2, GZ12 and GZ10 have high PV and FV values, which indicate that they have a high degree of solubility and the ability to form a strong gel [24,25]. Upon the cooling of the starch paste, the straight-chain starch reconnects, leading to starch degradation, which is also the cause of SV and SV reflects the extent to which sweetpotato hardens upon cooling [27]. The high SV values of GZ2 and GZ12 indicate that they have a high level of retrogradation. The BV values of SPH and J4 were lower than those of the other PFSPs, indicating that SPH and J4 have higher heat resistance and shear resistance [28]. The PT of the samples was 76.68–79.67 °C, of which the highest was SPH and the lowest was GZ1. The range of PT in this study was consistent with previous studies [29].

### 3.5. Sensory Evaluation

Differences in the qualities of different PFSPs can result in differences in their sensory evaluation. Compared with other PFSPs, GZ1 and G20 had more fibers and a noticeably coarse texture, which may result in a poor taste (Figure 2F). The starchiness of all PFSPs except GZ1 was high, and the highest was SPH. SPH had higher aroma scores, while GZ2 had the lowest aroma scores, indicating that the aroma of GZ2 was not obvious to the experimenters after steaming. Sweetness had a great influence on overall taste, with SPH being the sweetest of all PFSPs, followed by ZZ3, and the least sweet being GZ9, which had the highest anthocyanin content. Firmness was also an important indicator in the sensory evaluation. It can be seen from Figure 2F that GZ2 was the hardest, while J4 was the softest, and the difference between the other PFSPs was not large. Based on this sensory evaluation, in terms of overall taste score, SPH and ZZ3 had higher scores (both higher than 80 points), while GZ2 and GZ9 had lower scores (less than 70 points) and the remaining PFSPs’ scores were in the range of 70–80.

### 3.6. Correlation Network Analysis of Starch, Amylose, Soluble Sugar, Pasting Properties and Sensory Evaluation of PFSPs

In order to explain the role of quality in the sensory formation of PFSPs, we constructed a heat map of the correlation between the quality and sensory evaluation of steamed PFSPs (Figure 2G). The sensory evaluation of sweetpotatoes is mainly related to their amylose content, solubility and amylopectin structure, which affects the firmness and viscosity of sweetpotatoes [11,30]. Specifically, their dry matter content and total starch content were significantly positively correlated with firmness and negatively correlated with viscosity and smoothness. PFSPs with a high starch content had a dry and hard texture, which was consistent with previous research results [31,32]. Amylose was significantly positively correlated with sweetness, which may be due to the release of a large amount of amylose during starch pasting and the production of a large amount of soluble sugar [15]. The soluble sugar content was positively correlated with other sensory indexes, except firmness, and was significantly positively correlated with sweetness, which improved the overall taste of the PFSPs. The degree of the saccharification of starch during cooking determines the soluble sugar content, which affects its sweetness [12]. Anthocyanin content was negatively correlated with other sensory indexes except firmness. On the one hand, the bitterness of anthocyanins affects the sweetness of PFSPs [8]. The interaction between anthocyanins and starch may regulate pasting properties and lead to less starch degradation, which affects the taste of PFSPs [33]. The pasting properties were negatively correlated with most sensory indexes, and FV and SV were significantly negatively correlated with aroma, which may be due to the loss of aroma during cooling and the reassociation of starch. The soluble sugar content and amylose content were mainly positively correlated with the overall taste, while the anthocyanin content and pasting properties were negatively correlated with the overall taste, indicating that these qualities could affect the sensory evaluation of PFSPs. The overall taste was significantly positively correlated with aroma and sweetness, indicating that aroma and sweetness were the main indicators for evaluating the taste of PFSPs.

### 3.7. Volatile Metabolites Profiles

**Figure 3:**
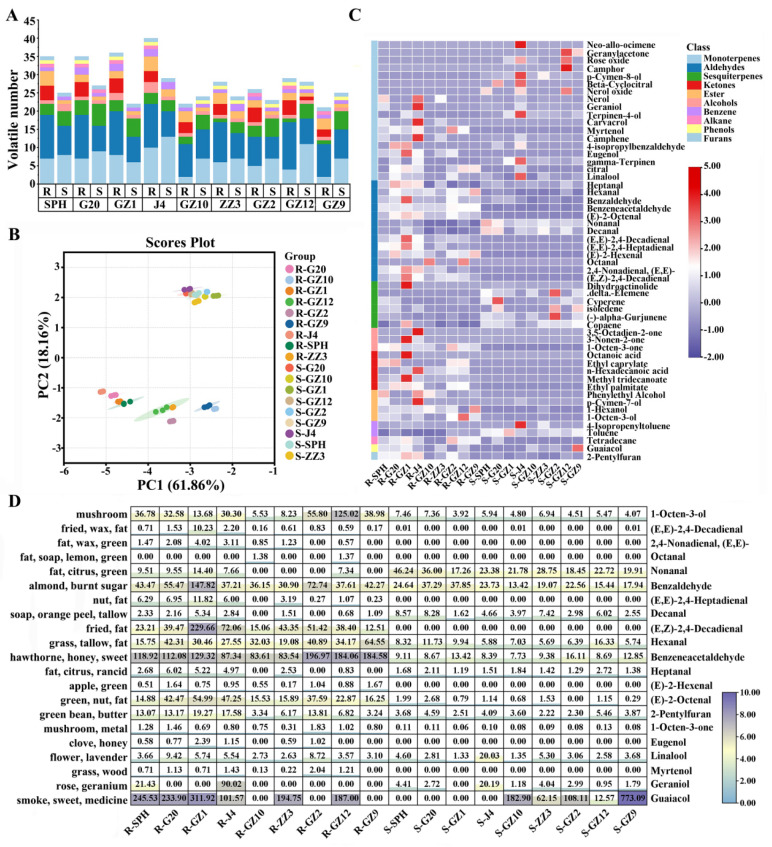
Analysis of volatile organic components in PFSPs before and after steaming. (**A**) Statistics of PFSPs’ volatile organic compounds before and after steaming. (**B**) PCA score plot; each point represents an independent experimental repetition. (**C**) Heat map visualization of all metabolites. The red squares represent up−regulated metabolites, the purple squares represent down−regulated metabolites and the white squares represent the average relative expression intensity of all volatile compounds. (**D**) ROAV diagram of different PFSPs.

In the process of thermal processing, starch and other substances may be degraded to form volatile organic compounds (VOCs), and the flavor formed by the volatile compounds of sweetpotatoes can directly affect the eating quality of sweetpotatoes [34]. The volatile metabolites of nine PFSPs before and after steaming were preliminarily identified by GC-MS, and a total of 54 VOCs were identified. The detailed information and relative content data of these 54 VOCs are shown in Table S2. Based on their chemical structure, the VOCs were divided into 10 different chemical classes: monoterpenes (18), aldehydes (13), sesquiterpenes (6), ester (5), alcohols (4), ketones (3), Benzene (2), alkane (1), Phenols (1) and Furans (1). Among them, aldehydes and terpenes (monoterpenes and sesquiterpenes) accounted for a large proportion of the VOCs in all samples (Figure 3A). A total of 35–40 VOCs were detected in the raw samples of light PFSPs, while only 21–29 VOCs were detected in the raw samples of deep PFSPs. The number of VOCs decreased in most PFSPs after steaming, except for GZ10 and GZ9. Eight VOCs were detected in all samples, and unique VOCs were detected in the raw and steamed samples of J4 (Figure S1), the raw samples of GZ1 and the steamed samples of GZ12.

The levels of VOC accumulation were used as the basis for performing a principal component analysis (PCA). The first and second principal components, PC1 and PC2, accounted for 61.86% and 18.16% of the variance, respectively, resulting in a cumulative explanation of 80.02% of the total variance (Figure 3B). All samples were negatively distributed on the PC1 axis, and there was no significant separation on the PC1 axis for the steamed samples of different varieties. However, for the raw sample, light PFSPs were separated from the rest of PFSPs, closer to the left side of the PC1 axis. GZ9 and GZ10 were closer to the right side of the PC1 axis than other PFSPs. On the PC2 axis, the raw samples showed a negative distribution, and the steamed samples showed a positive distribution, indicating that PC2 may respond to temperature-related indicators. The PCA results showed that the VOCs of PFSPs before and after steaming were clearly distinguished.

To understand the differences in VOC accumulation levels in different PFSPs varieties before and after steaming, we constructed a heat map (Figure 3C) based on their relative content and performed a one-way analysis of variance (Table S2). The heat map showed that there was a large difference between the raw and steamed samples of different sweetpotato varieties. The content of VOCs in the raw samples was about 2–8 times that in the steamed sample, which may be due to the escape of VOCs from the samples during the steaming process. Aldehydes and terpenes (monoterpenes and sesquiterpenes) were the most abundant VOCs in the samples (59–91%), and the accumulation levels of other VOCs were low. Aldehydes were the most abundant VOCs in the raw and steamed samples relative to each other. 

A total of 13 aldehydes were identified in this study and most of them decreased after steaming. Three aldehydes were detected in both raw and steamed samples, namely hexanal, benzaldehyde and phenylacetaldehyde, and their contents decreased after steaming. (E,E)-2,4-Heptadienal, (E)-2-Hexenal, Octanal, 2,4-Nonadienal, (E,E)- and (E,Z)-2,4-Decadienal were specifically detected only in the raw samples, and they had a plant-like aroma and a fatty flavor. Benzeneacetaldehyde from the degradation of the aromatic amino acid phenylalanine (Phe) and (E,E)-2,4-Decadienal from the auto-oxidation of oleic acid were more abundant VOCs in the raw samples, with a relative content more than 10 times that in the steamed samples [35]. The content of Nonanal (fat, citrus, green) and Decanal (soap, orange peel, tallow) increased after steaming. 

Terpenoids, including monoterpenoids and sesquiterpenoids, are important contributors to sweetpotatoes’ aroma. A total of 24 terpenes were detected in this study, including 18 monoterpenoids and 6 sesquiterpenoids (Figure 3C, Table S2). After steaming, except for J4, the content of monoterpenoids in the light PFSPs decreased, while, except for GZ2, the content of monoterpenoids in other deep PFSPs increased. J4 showed a significantly higher monoterpenoid content than the other PFSPs, and its content was about 3–8 times that of other PFSPs, but with lower levels of sesquiterpenoids. Linalool was the only monoterpenoid detected in all samples, with linalool levels increasing after steaming only in SPH, J4, ZZ3 and GZ9. Geraniol was higher in raw samples of J4 and SPH. Linalool and geraniol are formed from geranyl pyrophosphate by the action of terpene synthases or by the oxidation of myrcene, giving sweetpotatoes a floral aroma [36]. Geranylacetone, rose oxide, p-Cymen-8-ol, beta-Cyclocitral and nerol oxide were only specifically detected in steamed samples, which mainly brought herb and flower flavors to sweetpotatoes [37]. Except for GZ1, the sesquiterpenoid content of all PFSPs increased after cooking, which may be due to the low extraction temperature of the raw sample, which hindered its volatilization [34]. Only one sesquiterpenoid (delta-Elemene) was specifically detected in the steamed samples, providing them with a wood odor.

Esters may be formed in the process of the thermal degradation of glucose or the lipoxygenase degradation of fatty acids [34]. A total of five esters and four alcohols were detected in this study. All esters were detected only in the raw samples, except for ethyl palmitate, which was detected in the steamed GZ12 sample, possibly because the content of volatile esters decreases with increasing temperature [38]. In addition, three ketones were detected in this study. Among them, 3,5-octadiene-2-one was only specifically detected in J4 raw samples, which brought a pleasant fruity odor to J4 (Figures S2 and S3). 1-Octen-3-one (mushroom, metal) was detected in all samples, but its content was significantly reduced after steaming. Furan and its derivatives were often produced during the Maillard reaction and were important aroma contributors to the cooking of sweetpotatoes [39]. In this study, 2-pentylfuran was detected in all samples, and its content decreased significantly after steaming, which was consistent with previous research results [8]. Studying the changes in VOCs before and after the steaming of different varieties of PFSPs can provide a basis for selecting suitable PFSPs.

### 3.8. Odor Profiles of PFSPs

Although 54 VOCs were detected in the samples, only a fraction of the VOCs contributed significantly to the characteristic aroma of the PFSPs, and these were referred to as aroma-active compounds [40]. To evaluate the contribution of VOCs to the overall aroma, the rOAV was calculated based on the VOC data of the samples (Table S3, Figure 3D). A total of 21 VOCs in the raw samples had a rOAV > 1, while only 12 VOCs in the steamed samples had a rOAV > 1, which indicated that the aroma of the PFSPs was weakened after steaming. Most of the aroma-active compounds in all samples were aldehydes, indicating that most of the aroma of the PFSPs was composed of aldehydes [6]. The rOAV of (E,E)-2,4-decadienal was >1 in the raw samples of all light PFSPs except SPH, indicating that it contributes more to the aroma of the raw samples of light PFSPs, but a large amount of (E,E)-2,4-decadienal would bring an unpleasant smell to the samples [33]. Linalool had a rOAV > 1 in all samples, especially in the J4 steamed samples, where the rOAV was 2–14 times that of the other samples, providing a floral aroma to the PFSPs. In contrast, guaiacol had a rOAV > 1 only in the deep PFSPs’ steamed samples, especially in the GZ9 steamed samples, where its rOAV was as high as 773.09, which brought a tobacco flavor to the steamed samples of deep PFSPs.

## 4. Conclusions

In this study, the anthocyanins, starch, soluble sugar and VOCs of nine PFSPs before and after steaming were determined, and their differences were analyzed by combining their pasting properties and a sensory evaluation. The results showed that the anthocyanin content of deep PFSPs was also significantly higher than that of light PFSPs. After steaming, the anthocyanin content of some PFSPs was reduced, while the anthocyanin content of other PFSPs was increased, which was due to the increase in their anthocyanins’ recovery rate. Starch gelatinization during steaming resulted in a decrease in starch content and an increase in soluble sugar content in all samples, with J4 having the lowest starch content among the raw and steamed samples. However, in the raw samples, even though the soluble sugar contents of GZ12 and GZ9 were higher than those of ZZ3 and GZ10, their soluble sugar contents increased by a smaller amount during steaming due to less starch degradation taking place and possibly due to the effect of the anthocyanins. In the steamed samples, the soluble sugar content of the light PFSPs was significantly higher than that of the deep PFSPs. The pasting curves of all the deep PFSPs were higher than those of the light PFSPs, except for ZZ3. Among the nine PFSPs, SPH and ZZ3 had the best overall taste and higher sweetness and aroma scores. The relationship between the sweetpotatoes’ sensory evaluation and quality was analyzed using correlation heat maps. The results showed that soluble sugar content was positively correlated with aroma and sweetness, whereas anthocyanins and pasting properties were negatively correlated with most sensory indicators, which might be due to the interaction between anthocyanins and starch. In addition, 54 VOCs were identified in the PFSPs, with aldehydes and terpenes being the main VOCs in PFSPs. In the raw samples, the number of VOCs in the light PFSPs was larger than that in the deep PFSPs, but the number of VOCs was almost reduced after steaming. By calculating the rOAV, the results showed that aldehydes were the main aroma-active compounds of PFSPs.

This study comprehensively and systematically investigated the differences in the chemical composition and sensory evaluation of nine PFSPs before and after steaming, analyzed the correlation between quality and sensory evaluations and provided a reference for people to screen PFSPs for high nutritional value and a good taste.

## Figures and Tables

**Table 1:** Pasting properties of sweetpotato ^1^.

	PV (cP)	TV (cP)	BV (cP)	FV (cP)	SV (cP)	Peak Time (min)	PT (°C)
SPH	720.33 ± 6.11 g	525.33 ± 5.86 g	195.00 ± 1.00 f	786.33 ± 9.29 i	261.00 ± 3.46 g	4.84 ± 0.04 f	79.67 ± 0.49 a
G20	1909.00 ± 39.05 de	1518.00 ± 32.23 d	391.00 ± 9.17 d	2665.00 ± 58.89 e	1147.00 ± 27.07 c	6.07 ± 0.07 a	77.42 ± 0.49 d
GZ1	1963.33 ± 74.85 cd	1464.33 ± 79.22 d	499.00 ± 6.08 b	2467.00 ± 98.61 f	1011.67 ± 19.43 d	5.20 ± 0.00 e	76.68 ± 0.43 e
J4	1195.00 ± 39.00 f	1009.00 ± 25.00 f	186.00 ± 14.00 f	1688.00 ± 45.00 h	679.00 ± 20.00 f	5.17 ± 0.03 e	78.40 ± 0.05 c
GZ10	2311.33 ± 35.12 b	1866.67 ± 53.16 b	444.67 ± 39.70 c	3050.67 ± 43.75 c	1184.00 ± 28.93 c	5.64 ± 0.04 c	79.45 ± 0.39 a
ZZ3	1846.00 ± 44.80 e	1335.33 ± 30.86 e	510.67 ± 40.56 b	2297.67 ± 32.59 g	962.33 ± 20.43 e	4.89 ± 0.04 f	78.15 ± 0.48 c
GZ2	2345.00 ± 85.75 b	1992.00 ± 62.36 a	353.00 ± 23.39 d	3440.67 ± 94.82 a	1448.66 ± 32.58 a	5.58 ± 0.04 cd	79.18 ± 0.06 ab
GZ12	2501.67 ± 65.31 a	1928.33 ± 46.23 ab	573.33 ± 28.10 a	3234.33 ± 68.72 b	1306.00 ± 27.84 b	5.53 ± 0.000 d	78.68 ± 0.45 bc
GZ9	2024.00 ± 15.72 c	1758.67 ± 12.50 c	265.33 ± 21.08 e	2929.67 ± 5.13 d	1171.00 ± 9.64 c	5.93 ± 0.07 b	79.20 ± 0.05 ab

^1^ Data are means ± standard deviation. Values in the same column with different letters are significantly different (*p* < 0.05).

## Data Availability

The original contributions presented in the study are included in the article/Supplementary Materials, further inquiries can be directed to the corresponding author.

## Supplementary Materials

The following supporting information can be downloaded at https://www.mdpi.com/article/10.3390/foods13193168/s1, Table S1: sweetpotato evaluation standard; Table S2: identification of volatile compounds in nine purple-fleshed sweetpotatoes before and after steaming; Table S3: rOAVs in nine purple-fleshed sweetpotatoes before and after steaming; Figure S1: a TIC chromatogram of a raw sample of J4; Figures S2 and S3: EI mass spectra of 3,5-octadien-2-one.
