# Supplementary figures and images for: Effects of Steaming on Chemical Composition of Different Varieties of Purple-Fleshed Sweetpotato
Source: Foods. 2024 Oct 5;13(19):3168. doi: 10.3390/foods13193168 (PMC11475826; doi:10.3390/foods13193168)

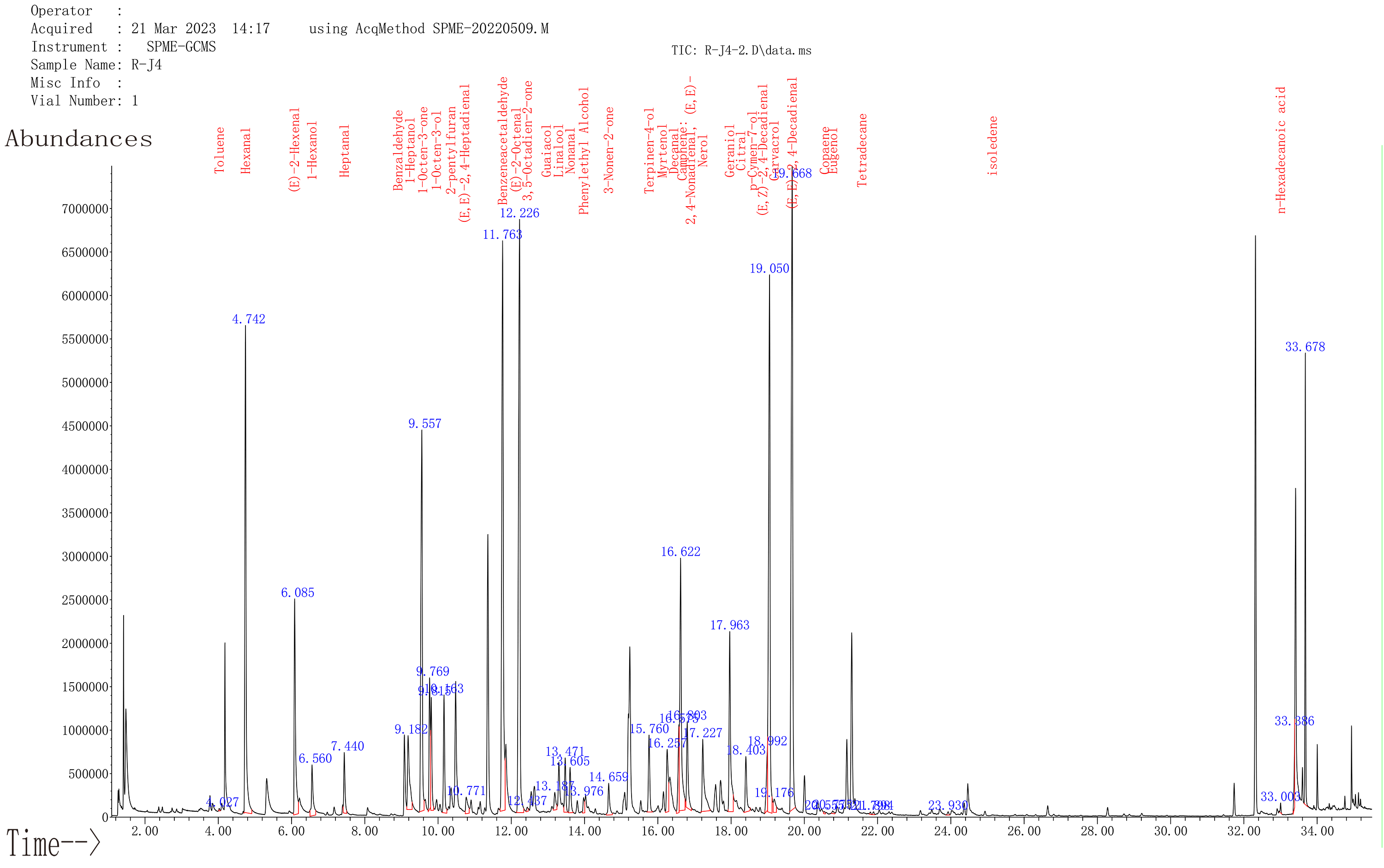

Supplement: Supplementary file 1 [file foods-13-03168-s001.zip › Figure S1.tif]

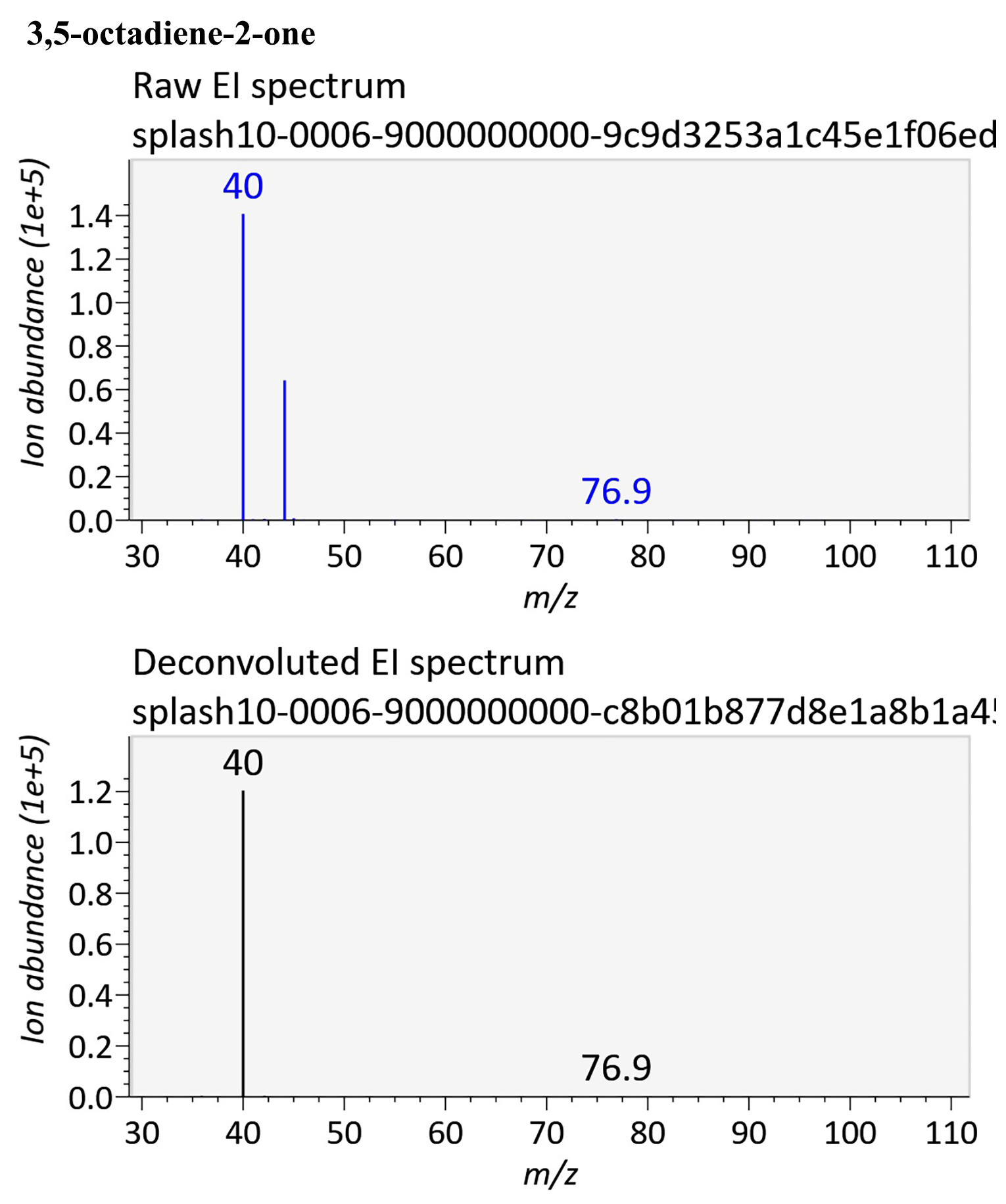

Supplement: Supplementary file 1 [file foods-13-03168-s001.zip › Figure S2.tif]

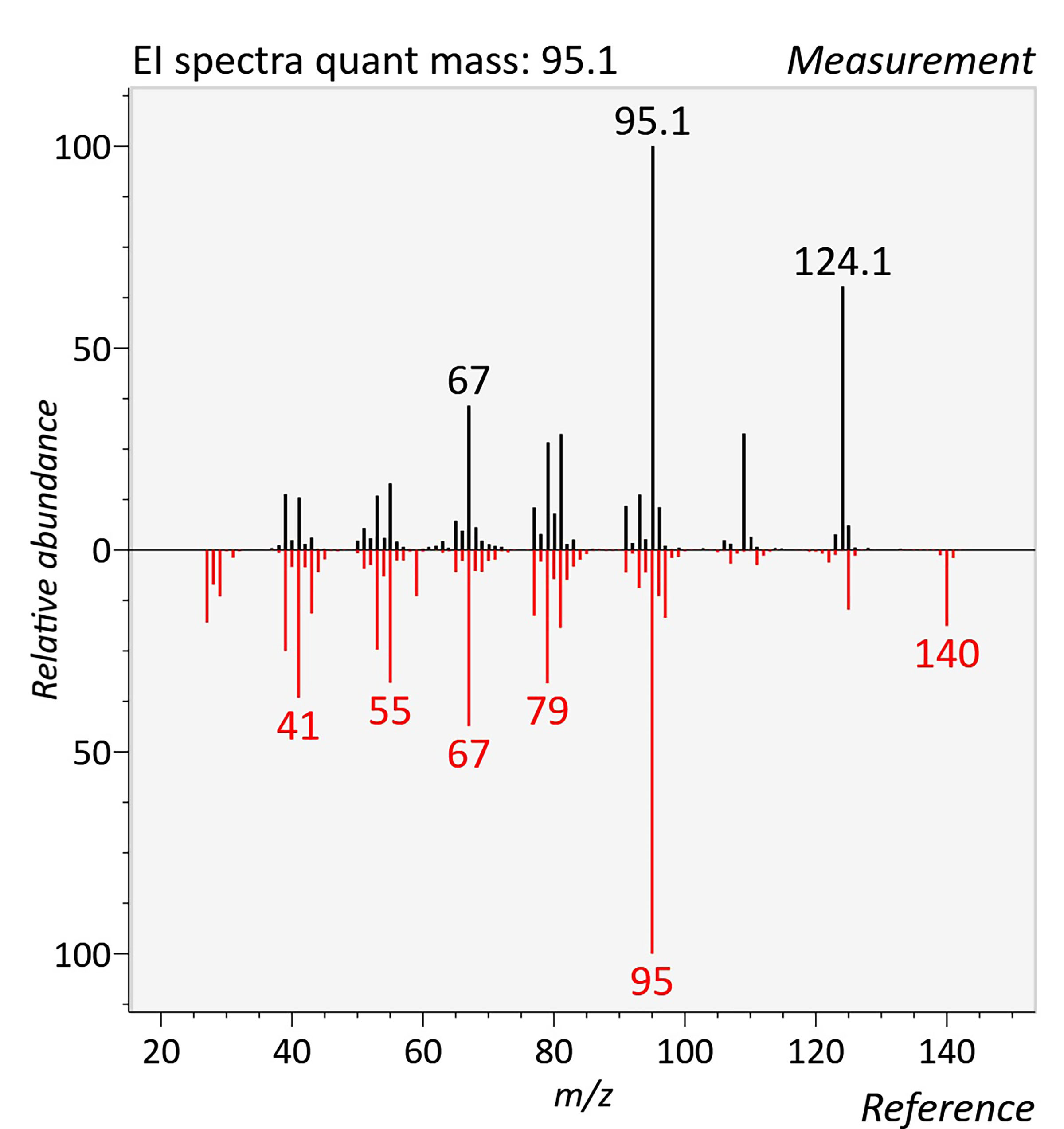

Supplement: Supplementary file 1 [file foods-13-03168-s001.zip › Figure S3.jpg]
